# Biomaterials and Electroactive Bacteria for Biodegradable Electronics

**DOI:** 10.3389/fmicb.2022.906363

**Published:** 2022-06-10

**Authors:** Robin Bonné, Koen Wouters, Jamie J. M. Lustermans, Jean V. Manca

**Affiliations:** ^1^Center for Electromicrobiology, Department of Biology, Aarhus University, Aarhus, Denmark; ^2^X-LAB, UHasselt, Diepenbeek, Belgium

**Keywords:** cable bacteria, long-range electron transport, bioelectronics, organic electronics, e-waste, e-biologics, microbial nanowires, biological semiconductor

## Abstract

The global production of unrecycled electronic waste is extensively growing each year, urging the search for alternatives in biodegradable electronic materials. Electroactive bacteria and their nanowires have emerged as a new route toward electronic biological materials (e-biologics). Recent studies on electron transport in cable bacteria—filamentous, multicellular electroactive bacteria—showed centimeter long electron transport in an organized conductive fiber structure with high conductivities and remarkable intrinsic electrical properties. In this work we give a brief overview of the recent advances in biodegradable electronics with a focus on the use of biomaterials and electroactive bacteria, and with special attention for cable bacteria. We investigate the potential of cable bacteria in this field, as we compare the intrinsic electrical properties of cable bacteria to organic and inorganic electronic materials. Based on their intrinsic electrical properties, we show cable bacteria filaments to have great potential as for instance interconnects and transistor channels in a new generation of bioelectronics. Together with other biomaterials and electroactive bacteria they open electrifying routes toward a new generation of biodegradable electronics.

## Introduction: E-Waste as a Global Problem

**FIGURE 1:**
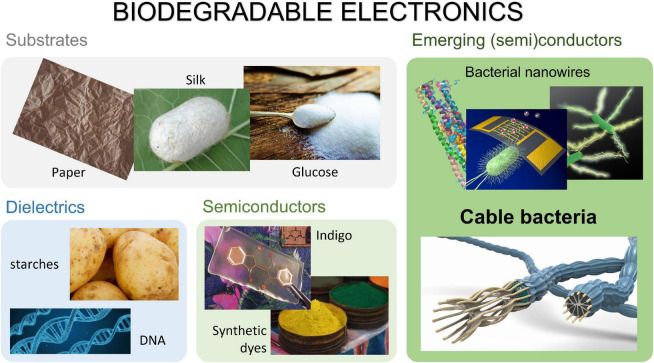
Several biomaterials that have potential in biodegradable electronics, electroactive bacteria and in particular cable bacteria are interesting upcoming candidates.

The use of electronic devices is creating the world’s fastest-growing waste-stream, with currently 50 million tons of e-waste produced each year (Ryder and Zhao Houlin, 2019). This not only causes $62.5 billion in material value of resources in our spent devices to be dumped into landfill (Ryder and Zhao Houlin, 2019), but exposure to this e-waste has a plethora of negative effects on humans, even *in utero* (Grant et al., 2013). Thus, electronic waste forms a substantial problem for environmental and human wellbeing, for which alternatives should be found on a short term (Kang et al., 2020). Biodegradable electronics is a new field that aims to replace the harmful non-durable materials used in electronics with biodegradable alternatives. In this review we will discuss the recent advances of using biomaterials for biodegradable electronics and the possible role of electroactive bacteria and in particular cable bacteria, of which an overview is given in Figure 1.

## Advances in Biodegradable Electronics

Biodegradability might seem like a broad term since all materials will be degraded when left in nature after enough time. We will focus on so-called transient electronics: materials that have limited lifetimes before they disappear with the ultimate goal being bioresorption: the complete degradation of a device when immersed in biofluids. The kinetics of biodegradable materials depend on their chemical and morphological properties and the environmental conditions, such as temperature and pH and ionic content when in a solution (Kang et al., 2014). The lifetime of these devices can be accurately controlled by encapsulating the device with biodegradable material or by actively triggering the degradation reaction (Hwang et al., 2012; Kang et al., 2014, 2020). The number of defects through which water/vapor—which degrade the device—could leak can be tuned multiple ways, for example, by using multiple layers (Kang et al., 2014). Another option is doping materials to hinder their electrolysis (Seidel et al., 1990).

Biodegradable electronics can be found among inorganic, organic, and biological materials. Transience by bioresorption has for example been simulated for (semi-) conductors such as Silicon (Si), Germanium (Ge), SiGe and amorphous indium gallium zinc oxide (a-IGZO) in aqueous media. Some proposed non-biological transient dielectric materials include metal or semiconductor oxides and nitrides such as MgO, SiO_2_, and SiN_x_ (Hwang et al., 2012). For example, the silicon and SiO_2_ undergo hydrolysis to form either harmless Si(OH)_4_ and H_2_, or Si(OH)_4_ and water (Seidel et al., 1990). This is a surface level effect, which allows for silicon’s use as encapsulation layer. Nevertheless, layers of SiO_2_ have defects through which water can permeate rapidly (<10 min), making it ineffective for such use (Lee et al., 2017). In this paper the focus will be on biodegradable materials originating from nature. They have the advantage that high-scale low-cost production is possible (Kang et al., 2020), because they are often solution based and allow low-temperature processing steps (Stadler et al., 2007). Furthermore, they have a high chemical compatibility with many other materials, and their properties can be tuned for their lifetime and morphology (Kang et al., 2020). Besides, organic monolayers can be combined with metals and plastics to create flexible electronics (Sekitani et al., 2009).

For biological biodegradable semiconductors, we will focus on materials that have proven conduction at least in the micrometer range, thereby excluding possible candidates like single peptides/proteins (Ing et al., 2018) and DNA (Beratan, 2019). A first interesting group of biological semiconductors are naturally occurring molecules such as indigo, which originates from several species of plants (Zollinger, 2003), but nowadays has become the most mass-produced industrial dye (Glowacki et al., 2011). These are planar molecules, which are connected through hydrogen bonding, resulting in tight π-stacking between neighboring molecules (Irimia-Vladu et al., 2012). This means that the orbitals which are not used in the intermolecular binding of the elements overlap and form conductive pathways. Despite having a high melting point, and low solubility, indigo can be degraded by enzymes (Campos et al., 2001), bacteria (Valdez-Vazquez et al., 2020), oxidizing agents (Prado et al., 2008) and light (in water) in timeframes down to a single day (Vautier et al., 2001), with a reported rate of 0.90 μmol L^–1^ min^–1^ (Vautier et al., 2001). In donor-acceptor solar cells, indigo acts like a good acceptor material (Glowacki et al., 2011). Another naturally occurring semiconductor is the pigment β-carotene, which can act as an electron donor in e.g., dye sensitized solar cells (Glowacki et al., 2011). However, this material is highly soluble in organic solvents, which makes certain measurements such as cyclic voltammetry challenging (Glowacki et al., 2011). Once dissolved it degrades with a reaction rate of 0.621 h^–1^ (Chen et al., 2014). The photo-to-electric conversion efficiency is very low when used in a dye sensitized solar cell (Suryana et al., 2013a). Eumelanins are a subclass of melanin which is found in skin pigment, which are natural semiconductors (Feig et al., 2018). Upon the absorption of water, a reaction occurs where free electrons and protons are released, self-doping the eumelanins. In 8 days, *in vivo* melanin implants can be nearly completely eroded and resorbed (Feig et al., 2018).

Instead of just considering naturally occurring biomolecules, it is interesting to look at their derivates to obtain organic semiconductors which are based on naturally occurring molecules as is reviewed by Glowacki et al. (2011). For example, indanthrene yellow G and indanthrene brilliant orange RK, are man-made pigments used in dyes that act as wide-bandgap semiconductors with high electron affinity. Other pigments can be derived from different sources to function as p-type electron donors like Quinacridone and Cibalakrot, with a larger bandgap (Glowacki et al., 2011). In 115 days, the conductivity of the former degraded to 80% of its original value (Daniel Głowacki et al., 2013). Gelatin is a conductive polymer obtained from collagen, of which the conductivity can be increased even further by combining it with conducting polymers such as Polyaniline (PANI) (Harrington and von Hippel, 1962; Li et al., 2006). Other biodegradable semiconductors which are neither natural nor nature-inspired include Poly(p-phenylene vinylene) (PPV), ambipolar polyselenophene, Buckminster fullerene, pentacene, and 5,50-bis-(7-dodecyl-9H-fluoren-2-yl)-2,20-bithiophene (DDFTTF).

Next to the biodegradable (semi) conducting materials, there is a much larger variety in isolating biomaterials that can be used as underlying substrates for the electronic circuitry, as described in Irimia-vladu et al. (2010b). The cheapest yet very familiar biodegradable substrate is paper. Despite its roughness, low-voltage active circuits can be realized on top of it using gravure and flexographic printing techniques. Hard gelatin capsules and caramelized glucose can be ingested and therefore used in bio-metabolizable electronics (Irimia-vladu et al., 2010b). Caramelized glucose is sensitive to moisture but has a smoothness close to that of glass. Poly(L-lactide-co-glycolide) (PLGA) are other sugar-based biopolymers obtained from pure L lactide, which can be used as substrate (Bettinger and Bao, 2010; Chanfreau et al., 2010). Though water can permeate through PLGA, which causes rapid failure (< 10 min) when they are used as encapsulation layer (Lee et al., 2017). Another familiar substrate is silk, a polypeptide polymer made up of fibroin and sericin, which have interchain hydrogen bonds, leading to the mechanical robustness of the silk fibers (Irimia-Vladu et al., 2012). Silk elicits no immune response and it safely dissolves and resorbs completely, making it safe to be implanted into the body. Furthermore, silk can be engineered to degrade under the desired conditions (Wang et al., 2008; Hu et al., 2011; Irimia-Vladu et al., 2012). A less common natural polymeric material produced by insects is shellac, a natural polyester copolymer, which can also be synthetically produced in multiple different grades and shades that can easily be cast to produce substrate foils (Irimia-Vladu et al., 2012). Of the mentioned substrates, glucose silk and shellac have very good surface smoothness. However, in general, biodegradable substrates need a smoothing layer to allow the fabrication of electronic components on top of them (Irimia-vladu et al., 2010b). An example of a material used for such a layer is polydimethylsiloxane (PDMS), which binds specific regions of the circuit to the substrate and provides strain isolation (Kim et al., 2009). The substrates described here are summarized with their dielectric constants and breakdown fields in Table 1.

**TABLE 1:** An overview for different (semi) conducting materials and substrate materials for biodegradable electronics with their relevant characteristics measured in dry conditions. Bacterial nanowires stand out against the known biodegradable semiconductors for their high conductivity and mobility values. Cable bacteria top other nanowires for their excessive conduction length and n-type transistor behavior.

**(Semi) Conducting materials**						
**Material**		**Cond. length (nanowires)**	**Conductivity (S/cm)**	**Transistor properties**	**Mobility (cm^2^/Vs)**	**References**

Biodegradable semiconductor	Indigo		10^−6^	n-type	0.01	Vautier et al., 2001; Bouzidi et al., 2017
	B-carotene		10^−3^	p-type	0.0004	Suryana et al., 2013b
	Eumelanin		10^−5^	n-type	0.01	Ambrico et al., 2011; di Mauro et al., 2021
	Quinacridone		10^−4^	p-type	0.001	Inokuchi and Akamatu, 1961; Enengl et al., 2015
	Perylene diimide		10^−1^ (Doped, redox cond.)	n-type	0.01	Gregg and Cormier, 1998
Polymer nanowire	P3HT	10 μm	10 (Doped)	p-type	0.1	Briseno et al., 2008; Paterson et al., 2018
	PEDOT:PSS	10 μm	10	p-type	10	Groenendaal et al., 2000; Wei et al., 2013
Bacterial nanowire (wild type)	*Geobacter sulfurreducens*	10 μm	5	p-type	0.03	Reguera et al., 2005; Lampa-Pastirk et al., 2016
	*Shewanella oneidensis*	10 μm	1	p-type	0.1	El-Naggar et al., 2010; Leung et al., 2013
	Cable bacteria	1 cm	10	n-type	0.1	Meysman et al., 2019; Bonné et al., 2020

**Substrate materials**						

**Material**		**Dielectric constant**		**Breakdown field (MV/cm)**		**References**

Paper (with oil)		2.3		0.3		Feng et al., 2020
Caramelized glucose		6.4		1.5		Irimia-vladu et al., 2010a
Silk		2.7		2.5		Townsend et al., 1997
Silk (treated)		4.6		4.0		Dickerson et al., 2013
Shellac		3.1		8		Irimia-Vladu et al., 2013
Ecoflex^®^		2.2		0.2		Siegenthaler et al., 2012b

After looking at plant and animal based biodegradable organic substrates, one can look for substrates originating from microorganisms, an example of which is poly(4hydroxybutyrate) (P4HB), as described by Martin and Williams (2003) and Bettinger and Bao (2010). This material is produced by genetically engineered microorganisms, usually *Escherichia coli* K12, since it is very difficult to produce polymers fit for most applications through chemical synthesis (Martin and Williams, 2003). P4HB is strong, with a tensile strength comparable to high molecular weight polyethylene, yet flexible, with an elongation to break of 1,000% and is often extremely well tolerated *in vivo* (Martin and Williams, 2003). As for the semiconductors, there are biodegradable substrates which are neither naturally occurring nor nature inspired. For example, Ecoflex^®^ is a completely biodegradable aliphatic-aromatic polyester which combines excellent mechanical properties, such as its tear-resistance, flexibility, and its resistance to fluctuations in water and humidity, with good processability of synthetic thermoplastics (Siegenthaler et al., 2012a). It is certified worldwide as compostable and approved for contact with food, however, this is only compostable in an industrial composter. A distinction is made between Ecoflex^®^ F, based on fossil monomers, and Ecoflex^®^ FS, a compound with poly(lactic acid) that has a higher rate of biodegradation. The material can also be blended with poly(lactic acid) (PLA), to obtain a blend called Ecovio.

Other materials crucial in the production of capacitors and advanced electrical components like Field-Effect Transistors (FETs) are dielectrics. These are electrical insulators which can be polarized by electric fields such as the previously mentioned biodegradable substrates, but also less sturdy materials. An abundant example is DNA, which can be obtained for example as waste material from the fishing industry and processed from water-based solutions (Irimia-Vladu et al., 2012). When DNA is used in an Organic Field-Effect Transistor (OFET), it displays significant hysteresis in the transfer characteristics, because of its high permittivity (Stadler et al., 2007). The separate nucleobases which compose DNA, adenine, cytosine, guanine, thymine, and uracil (which replaces thymine in RNA) could be used instead. These molecules are abundant and have low toxicity and cost (Irimia-vladu et al., 2010b). Another kind of biodegradable dielectric material are starches (Siegenthaler et al., 2012a). They consist of amylose and amylopectin polymers, forming a multilayer structure through hydrogen bonding. Starches obtained from different types of plants, such as corn or potato, differ, having an influence on the particle size and moisture absorption. Pure starch has multiple properties which makes it difficult to process and does not give it many applications. However, it is possible to chemically modify the starch to overcome these problems, though not enough to use it as substrate (Siegenthaler et al., 2012a). However, it can be used as such if the starch is blended with a hydrophobic polymer such as Ecoflex^®^.

## Electroactive Bacteria for Bioelectronics

In the last decades, other extraordinary electronic materials were found in the world of microbiology: electroactive bacteria that make their own conductive wires. These electroactive microbes developed the ability to respire through minerals or other organisms (Summers et al., 2010; Shi et al., 2016; Sure et al., 2016), either through electron shuttles or as direct contact through self-made nanowires that form an electrical bridge between the bacterium and the mineral. When studying these bacteria in a bioelectrochemical system (BES), they were found to also respire through electrodes. The chosen conditions of the BES, like available substrates and electric potential of the electrode, can be tuned to enhance the nanowire production (Liu and Li, 2020; Yalcin et al., 2020). These bacterial nanowires are found in many forms and debate is still going on about its exact function and chemical structure (Creasey et al., 2018). Below we give an overview of nanowires that were found to be electrically conductive after isolation, both in wet and dry environments (Ing et al., 2018).

One organism intensively studied for its protein nanowires is *Geobacter sulfurreducens* (Reguera et al., 2005). Nanowires are often found in the form of c-type cytochromes: a heme containing protein with a Fe atom at its core. The prominent example is a nanowire made of a coiling stack of the cytochrome OmcS that consists of 6 heme groups (Wang et al., 2019) with measured conductivities in the order of 5 mS cm^–1^ (Malvankar et al., 2011; Yalcin et al., 2020). Another cytochrome nanowire made from 8-heme OmcZ proteins even reaches conductivities that are 1,000-fold higher (Yalcin et al., 2020). Next to cytochrome nanowires, a nanowire based on PilA proteins (nicknamed e-pili) have been described in many works (Reguera et al., 2005). Although controversy persists, there is ample evidence of these nanowires conducting electricity with conductivities in the range of 50–200 mS cm^–1^, respectively, for pH 7 and pH 2 (Adhikari et al., 2016). Another study found conductivities in the range of 1–5 S cm^–1^ with estimated mobilities in the range of 0.02 cm^2^ V^–1^ s^–1^ (Lampa-Pastirk et al., 2016). In another well-studied organism called *Shewanella oneidensis*, nanowires are found in the form of extensions of the outer membrane (Gorby et al., 2006; Pirbadian et al., 2014) that are packed with 20-heme protein complexes made of MtrA, MtrB, MtrC and OmcA cytochromes. These nanowires reach values of up to 1 S cm^–1^ (El-Naggar et al., 2010) and appear to have p-type transistor behavior with mobility values in the range of 0.1 cm^2^ V^–1^ s^–1^ (Leung et al., 2013). Besides these organisms a whole zoo of over 100 different electroactive bacteria (Logan et al., 2019) and their nanowires are either confirmed to have similar properties (Walker et al., 2018, 2019) or are waiting to be tested for their electronic potential.

Even though we can find some very good electrical characteristics for these nanowires, modifications to the wild-type nanowires can boost the conductivity values even further. The introduction of the *PilA* gene from *Geobacter metallireducens* in *G. sulfurreducens*, e.g., produces e-pili with conductivities in the order of 300 S cm^–1^ (Tan et al., 2017), while the inclusion of more aromatic rings like tryptophan in e-pili increased the conductivities to 100 S cm^–1^ at pH 7 and 1,000 S cm^–1^ at pH 2 (Tan et al., 2016). When thinking about applying these bacterial nanowires in biodegradable electronics, one might assume an intensive and costly process, but the recent discovery of nanowire production through genetic manipulation of *Escherichia coli* (Ueki et al., 2020) and the development of bottom-up fabrication of e-pili (Cosert et al., 2019) suggest a means of easier and cheaper production in the long term. Functionalization and adhesion of different substrates is also made easier with the possibility of decorating nanowires with peptides (Ueki et al., 2019). First proof-of-principle applications are developed as sensors (Smith et al., 2020), (limited) electricity production (Liu et al., 2020) and they are expected to play a role as conductors in bioelectronics (Lovley and Yao, 2021).

## A Special Case: Cable Bacteria for Biodegradable Electronics

The main drawback for bacterial nanowires is the relatively short range of conduction, as they were only constructed over micrometer distances for now (Ing et al., 2018). Ten years ago, a new bacterium with the ability of transporting electrons over centimeter distances was discovered in the sea sediment: Cable bacteria (Pfeffer et al., 2012). They developed this mechanism to reach for both H_2_S in the deeper layer of the sediment and the O_2_ that is only available at the surface. Coupling the redox reactions of sulfide oxidation and oxygen reduction (Nielsen et al., 2010), it was hypothesized that cable bacteria could transport electrical currents over their centimeter long bodies (Pfeffer et al., 2012). This hypothesis appeared true when Meysman et al. (2019) measured conduction through dry filaments of cable bacteria and found it to be highly conductive with conductivities ranging in the order of 0.01–10 S cm^–1^, with one measurement even reaching 79 S cm^–1^. The current was shown to be flowing in an organized structure of around 10–60 parallel wires of each 50 nm in diameter that are present just under the outer membrane of the bacterium (Cornelissen et al., 2018) and that are interconnected at cell junctions (Thiruvallur Eachambadi et al., 2020).

The results presented in previous works show cable bacteria can overcome the micrometer range conduction previously present in electromicrobiology, as conduction has been shown over more than 1 cm, thereby proving by far the longest electron transport in a single biological structure (Meysman et al., 2019). One use of the conductive fibers in cable bacteria could be as an interconnector in a circuit, as they show low contact resistance (Bonné et al., 2020) and conductivities exceeding 10 S cm^–1^ (Meysman et al., 2019). The typical currents found *in vivo* are in the order of 0.1–1 nA for a single filament (Pfeffer et al., 2012; Schauer et al., 2014), but measurements on the conductive fiber sheaths show that single filaments can bear currents up to 0.3 μA at a DC bias of 1V (Bonné et al., 2020), and even higher values could be expected. This corresponds with a current density of 3 A mm^–2^, comparable to the typical allowed density in household copper wires (5–20 A mm^–2^). Next to its interconnection properties, it was shown that cable bacteria could work as transistors with FET mobilities in the range of 0.1 cm^2^ V^–1^ s^–1^ (Bonné et al., 2020), giving them a potential role in future computational circuits. Finally, next to direct current (DC), alternating current (AC) signals have been shown to pass through cable bacteria (Bonné et al., 2020). Showing visible degradation after 9–30 days in its near-natural environment during which time it has been exposed to mainly (micro-) oxic conditions (Supplementary Figure 1), this suggests that cable bacterium could be an interesting biodegradable material for bioelectronics.

To put the values found for cable bacteria in perspective, in Table 1 their initial electronic properties are compared to the previously discussed biodegradable semiconductors. Conductivity, mobility, and transistor properties for these materials were obtained by dry solid-state measurements like current-voltage-analysis, atomic force microscopy and field-effect transistor measurements (doped perylene diimide, treated in an electrochemical setup, being the only exception). It is clear that cable bacteria stand out with their high mobility values and conductivities that are a few orders of magnitude larger. Next to that, Table 1 also shows the state-of-the-art characteristics of the model (wild type) microbial nanowires of *G. sulfurreducens* and *S. oneidensis* and well-studied organic nanowires P3HT and PEDOT:PSS, also resulting from dry solid state measurements. It is apparent that already after a few years of measurements, cable bacteria can easily compete with materials that are studied for one or a few decades. Conductivity and mobility values of cable bacteria fibers can be found in the same range as organic electronics and wild type nanowires, with PEDOT:PSS outrunning alternatives in terms of mobility. However, cable bacteria really stand out for their n-type transistor behavior that is uncommon for biological materials and even more for the conduction length that is a few orders of magnitude higher than typical lengths for other biological and organic nanowires.

## Conclusion, Challenges, and Outlook

The increasing amount of e-waste that is created worldwide has put an urge on the search for biodegradable electronics. In the mini-review presented in this work, we found that important progress was made in finding more sustainable materials in electronics, but their limited applicability in modern day devices shows they have a long way to go. An important element missing in most studies so far is a common and quantitative study on the rate and conditions of biodegradability of the different materials, which is crucial for their further investigation as bioelectronic materials. In the search for biological materials that could function as conductors or transistors in biodegradable electronics, interesting candidates are found in the world of electromicrobiology (Lovley, 2012), where electroactive bacteria like *Geobacter* and *Shewanella* species produce nanowires as electron transport conduits. Recent progress in the study of different proteins (Yalcin et al., 2020) and genetic manipulation has ramped up the conductivity values (Tan et al., 2016, 2017); modification of *Escherichia coli* has made PilA based nanowires easy to produce (Ueki et al., 2020). Yet, conduction lengths exceeding micrometers are yet to be shown, as well as the implementation in biodegradable electronic circuitry. An answer to this shortcoming might be cable bacteria that create nanowire networks that show electron transport over centimeters with high conductivities and electron mobility values.

Although the characteristics in Table 1 seem promising, research on cable bacteria is only in a young stage, and some limitations must be overcome to meet industrial requirements. Firstly, values for the conductivity can vary a few orders of magnitude among experiments, with Table 1 only displaying the order or magnitude of the highest measured values. This might be because all measurements were done on different species of cable bacteria taken from natural sediments that come from different field sites (Kjeldsen et al., 2019) and the variability found among cells within one organism (Bonné et al., 2020). Moreover, the measurement conditions were different for all filaments. As current through a bacterium decays exponentially in ambient air, a sample preparation time that takes 1 min longer (before placing the sample in a nitrogen environment or vacuum) might change the conductivity by a factor of 2. Although the true conductivity of the conductive fibers in cable bacteria is expected to be close to and possibly higher than the maximally reported value of 79 S cm^–1^, a more consistent set of values of the electronic properties is needed for the different species of cable bacteria. As a second limitation, all species of cable bacteria lack a pure culture so far, making upscaling difficult. Although some attempts have been made toward it, only a single-strain sediment culture was achieved until this point (Thorup et al., 2021). Finally, the conductive periplasmic fibers are yet to be extracted properly from cable bacteria, as it is expected that the pure fibers rather than the full filaments will be used in future electronics (Cosert et al., 2019). An easy and cheap method must be developed to either fully extract the fibers or produce them in a laboratory environment. It has to be emphasized that the chemical nature of the conductive fibers in cable bacteria is not yet discovered (Kjeldsen et al., 2019; Boschker et al., 2021; Thiruvallur Eachambadi et al., 2021), making it difficult to describe the electron transport mechanism, quantify the biodegradability of the material, and create a detailed forecast of the applicability in bioelectronics.

As a general conclusion, it can be stated that significant progress is being made toward biodegradable electronics, in terms of the usage and development of biomaterials and electroactive bacteria. Within the class of electroactive bacteria, cable bacteria show some very interesting features that could make them a potential long-range biodegradable interconnector or transistor in future durable electronics. Although the research field needs to overcome a few challenges before being able to get scaled up, the bacteria were only discovered 10 years ago and only in 2019 the first direct electrical measurements were made. If this blooming trend continues as it did for bacterial nanowires (Lovley and Walker, 2019), the road is open for cable bacteria fibers or cable bacteria inspired materials to be integrated together with a variety of complementary biomaterials and electroactive bacteria in our electronics in another 10 years from now. This new generation of electronics—being biodegradable—will dissolve in time together with the global e-waste problem.

## Author Contributions

JM conceived the study. RB and KW wrote the manuscript with equal contribution. JL made Supplementary Figure 1. All authors did editing and proofreading.

## Conflict of Interest

The authors declare that the research was conducted in the absence of any commercial or financial relationships that could be construed as a potential conflict of interest.

## Publisher’s Note

All claims expressed in this article are solely those of the authors and do not necessarily represent those of their affiliated organizations, or those of the publisher, the editors and the reviewers. Any product that may be evaluated in this article, or claim that may be made by its manufacturer, is not guaranteed or endorsed by the publisher.

## References

[B1] Adhikari R. Y. Malvankar N. S. Tuominen M. T. Lovley D. R. (2016). Conductivity of individual Geobacter pili. *RSC Adv.* 6 8354–8357. 10.1039/c5ra28092c

[B2] Ambrico M. Ambrico P. F. Cardone A. Ligonzo T. Cicco S. R. di Mundo R. (2011). Melanin layer on silicon: An attractive structure for a possible exploitation in bio-polymer based metal-insulator-silicon devices. *Adv. Mat.* 23 3332–3336. 10.1002/ADMA.201101358 21671448

[B3] Beratan D. N. (2019). Why Are DNA and Protein Electron Transfer So Different? *Ann. Rev. Phys. Chem.* 70 71–97. 10.1146/annurev-physchem-042018-052353 30726186 PMC6591729

[B4] Bettinger C. J. Bao Z. (2010). Organic thin-film transistors fabricated on resorbable biomaterial substrates. *Adv. Mat.* 22 651–655. 10.1002/adma.200902322 20217767 PMC2868598

[B5] Bonné R. Hou J. L. Hustings J. Wouters K. Meert M. Hidalgo-Martinez S. (2020). Intrinsic electrical properties of cable bacteria reveal an Arrhenius temperature dependence. *Sci. Rep.* 1:19798. 10.1038/s41598-020-76671-5 33188289 PMC7666173

[B6] Boschker H. T. S. Cook P. L. M. Polerecky L. Eachambadi R. T. Lozano H. Hidalgo-Martinez S. (2021). Efficient long-range conduction in cable bacteria through nickel protein wires. *Nat. Commun.* 12:3996. 10.1038/s41467-021-24312-4 34183682 PMC8238962

[B7] Bouzidi A. Yahia I. S. El-Sadek M. S. A. (2017). Novel and highly stable indigo (C.I. Vat Blue I) organic semiconductor dye: Crystal structure, optically diffused reflectance and the electrical conductivity/dielectric behaviors. *Dyes Pigments* 146 66–72. 10.1016/J.DYEPIG.2017.06.046

[B8] Briseno A. L. Mannsfeld S. C. B. Jenekhe S. A. Bao Z. Xia Y. (2008). Introducing organic nanowire transistors. *Materials Today* 11 38–47. 10.1016/S1369-7021(08)70055-5

[B9] Campos R. Kandelbauer A. Robra K. H. Cavaco-Paulo A. Gübitz G. M. (2001). Indigo degradation with purified laccases from Trametes hirsuta and Sclerotium rolfsii. *J. Biotechnol.* 89 131–9. 10.1016/S0168-1656(01)00303-0 11500206

[B10] Chanfreau S. Mena M. Porras-Domínguez J. R. Ramírez-Gilly M. Gimeno M. Roquero P. (2010). Enzymatic synthesis of poly-L-lactide and poly-L-lactide-co-glycolide in an ionic liquid. *Bioproc. Biosyst. Eng.* 33 629–638. 10.1007/s00449-009-0388-8 19888606

[B11] Chen L. Bai G. Yang R. Zang J. Zhou T. Zhao G. (2014). Encapsulation of β-carotene within ferritin nanocages greatly increases its water-solubility and thermal stability. *Food Chem.* 149 307–312. 10.1016/J.FOODCHEM.2013.10.115 24295711

[B12] Cornelissen R. Bøggild A. Thiruvallur Eachambadi R. Koning R. I. Kremer A. Hidalgo-Martinez S. (2018). The Cell Envelope Structure of Cable Bacteria. *Front. Microbiol.* 9:3044. 10.3389/fmicb.2018.03044 30619135 PMC6307468

[B13] Cosert K. M. Castro-Forero A. Steidl R. J. Worden R. M. Reguera G. (2019). Bottom-up fabrication of protein nanowires *via* controlled self-assembly of recombinant Geobacter pilins. *mBio* 10:e02721–19 10.1128/mBio.02721-19 31822587 PMC6904877

[B14] Creasey R. C. G. Mostert A. B. Nguyen T. A. H. Virdis B. Freguia S. Laycock B. (2018). Microbial nanowires – Electron transport and the role of synthetic analogues. *Acta Biomat.* 69 1–30. 10.1016/j.actbio.2018.01.007 29357319

[B15] Daniel Głowacki E. Irimia-Vladu M. Kaltenbrunner M. Ga J. White M. S. Monkowius U. (2013). Hydrogen-Bonded Semiconducting Pigments for Air-Stable Field-Effect Transistors. *Adv. Materials* 25 1563–1569. 10.1002/ADMA.201204039 23239229

[B16] di Mauro E. Rho D. Santato C. (2021). Biodegradation of bio-sourced and synthetic organic electronic materials towards green organic electronics. *Nat. Commun.* 1:3167. 10.1038/s41467-021-23227-4 34039966 PMC8154894

[B17] Dickerson M. B. Fillery S. P. Koerner H. Singh K. M. Martinick K. Drummy L. F. (2013). Dielectric breakdown strength of regenerated silk fibroin films as a function of protein conformation. *Biomacromolecules* 14 3509–3514. 10.1021/BM4008452 23987229

[B18] El-Naggar M. Y. Wanger G. Leung K. M. Yuzvinsky T. D. Southam G. Yang J. (2010). Electrical transport along bacterial nanowires from Shewanella oneidensis MR-1. *Proc. Natl. Acad. Sci.* 107 18127–18131. 10.1073/pnas.1004880107 20937892 PMC2964190

[B19] Enengl C. Enengl S. Havlicek M. Stadler P. Glowacki E. D. Scharber M. C. (2015). The Role of Heteroatoms Leading to Hydrogen Bonds in View of Extended Chemical Stability of Organic Semiconductors. *Adv. Funct. Mat.* 25 6679–6688. 10.1002/ADFM.201503241

[B20] Feig V. R. Tran H. Bao Z. (2018). Biodegradable Polymeric Materials in Degradable Electronic Devices. *ACS Central Sci.* 4 337–348. 10.1021/acscentsci.7b00595 29632879 PMC5879474

[B21] Feng D. Hao J. Yang L. Liao R. Chen X. Li J. (2020). Comparison of AC breakdown characteristics on insulation paper (pressboard) immersed by three-element mixed insulation oil and mineral oil. *High Volt.* 5 298–305. 10.1049/HVE.2019.0103 35526269

[B22] Glowacki E. D. Leonat L. Voss G. Bodea M. Bozkurt Z. Irimia-Vladu M. (2011). Natural and nature-inspired semiconductors for organic electronics. *Organic Semiconductors in Sensors and Bioelectronics IV* (Bellingham:SPIE) M1–M81180. 10.1117/12.892467

[B23] Gorby Y. A. Yanina S. McLean J. S. Rosso K. M. Moyles D. Dohnalkova A. (2006). Electrically conductive bacterial nanowires produced by Shewanella oneidensis strain MR-1 and other microorganisms. *Proc. Natl. Acad. Sci. U.S.A.* 103 11358–11363. 10.1073/pnas.0604517103 16849424 PMC1544091

[B24] Grant K. Goldizen F. C. Sly P. D. Brune M. N. Neira M. van den Berg M. (2013). Health consequences of exposure to e-waste: A systematic review. *Lancet Glob. Health* 1 e350–e361. 10.1016/S2214-109X(13)70101-3 25104600

[B25] Gregg B. A. Cormier R. A. (1998). Liquid Crystal Perylene Diimide Films Characterized by Electrochemical, Spectroelectrochemical, and Conductivity versus Potential Measurements. *J. Phys. Chem. B* 102 9952–9957. 10.1021/JP982842E

[B26] Groenendaal L. Jonas F. Freitag D. Pielartzik H. Reynolds J. R. (2000). Poly(3,4-ethylenedioxythiophene) and Its Derivatives: Past, Present, and Future. *Adv. Mat.* 12 481–494. 10.1002/(SICI)1521-4095(200004)12:7<481::AID-ADMA481<3.0.CO;2-C

[B27] Harrington W. F. von Hippel P. H. (1962). The Structure Of Collagen And Gelatin. *Adv. Protein Chem.* 16 1–138. 10.1016/S0065-3233(08)60028-5 13952907

[B28] Hu X. Shmelev K. Sun L. Gil E. S. Park S. H. Cebe P. (2011). Regulation of silk material structure by temperature-controlled water vapor annealing. *Biomacromolecules* 12 1686–1696. 10.1021/bm200062a 21425769 PMC3090511

[B29] Hwang S.-W. Tao H. Kim D.-H. Cheng H. Song J.-K. Rill E. (2012). A Physically Transient Form of Silicon Electronics. *Science* 337 1640–1644. 10.1126/science.1226325 23019646 PMC3786576

[B30] Ing N. L. El-Naggar M. Y. Hochbaum A. I. (2018). Going the Distance: Long-Range Conductivity in Protein and Peptide Bioelectronic Materials. *J. Phys. Chem. B* 122 10403–10423. 10.1021/acs.jpcb.8b07431 30240221

[B31] Inokuchi H. Akamatu H. (1961). Electrical Conductivity of Organic Semiconductors. *Solid State Phys.Adv. Res. Appl.* 12 93–148. 10.1016/S0081-1947(08)60653-0

[B32] Irimia-vladu B. M. Troshin P. A. Reisinger M. Shmygleva L. Kanbur Y. Schwabegger G. (2010a). Biocompatible and Biodegradable Materials for Organic Field-Effect Transistors. 4069–4076. 10.1002/adfm.201001031

[B33] Irimia-vladu M. Troshin P. A. Reisinger M. Schwabegger G. Ullah M. Schwoediauer R. (2010b). Environmentally sustainable organic field effect transistors. *Organic Elect.* 11 1974–1990. 10.1016/j.orgel.2010.09.007

[B34] Irimia-Vladu M. Głowacki E. D. Schwabegger G. Leonat L. Akpinar H. Z. Sitter H. (2013). Natural resin shellac as a substrate and a dielectric layer for organic field-effect transistors. *Green Chem.* 15 1473–1476. 10.1039/C3GC40388B

[B35] Irimia-Vladu M. Głowacki E. D. Voss G. Bauer S. Sariciftci N. S. (2012). Green and biodegradable electronics. *Materials Today* 15 340–346. 10.1016/S1369-7021(12)70139-6

[B36] Kang S. K. Hwang S. W. Cheng H. Yu S. Kim B. H. Kim J. H. (2014). Dissolution behaviors and applications of silicon oxides and nitrides in transient electronics. *Adv. Funct. Materials* 24 4427–4434. 10.1002/adfm.201304293

[B37] Kang S. K. Yin L. Bettinger C. (2020). The emergence of transient electronic devices. *MRS Bull.* 45 87–95. 10.1557/mrs.2020.19

[B38] Kim D. H. Kim Y. S. Wu J. Liu Z. Song J. Kim H. S. (2009). Ultrathin silicon circuits with strain-isolation layers and mesh layouts for high-performance electronics on fabric, vinyl, leather, and paper. *Adv. Materials* 21 3703–3707. 10.1002/adma.200900405

[B39] Kjeldsen K. U. Schreiber L. Thorup C. A. Boesen T. Bjerg J. T. Yang T. (2019). On the evolution and physiology of cable bacteria. *Proc. Natl. Acad. Sci.* 116:201903514. 10.1073/pnas.1903514116 31427514 PMC6754541

[B40] Lampa-Pastirk S. Veazey J. P. Walsh K. A. Feliciano G. T. Steidl R. J. Tessmer S. H. (2016). Thermally activated charge transport in microbial protein nanowires. *Sci. Rep.* 6:23517. 10.1038/srep23517 27009596 PMC4806346

[B41] Lee Y. K. Yu K. J. Song E. Barati Farimani A. Vitale F. Xie Z. (2017). Dissolution of Monocrystalline Silicon Nanomembranes and Their Use as Encapsulation Layers and Electrical Interfaces in Water-Soluble Electronics. *ACS Nano* 11 12562–12572. 10.1021/acsnano.7b06697 29178798 PMC5830089

[B42] Leung K. M. Wanger G. El-Naggar M. Y. Gorby Y. Southam G. Lau W. M. (2013). Shewanella oneidensis MR-1 Bacterial Nanowires Exhibit p-Type, Tunable Electronic Behavior. *Nano Lett.* 13 2407–2411. 10.1021/nl400237p 23701405

[B43] Li M. Guo Y. Wei Y. MacDiarmid A. G. Lelkes P. I. (2006). Electrospinning polyaniline-contained gelatin nanofibers for tissue engineering applications. *Biomaterials* 27 2705–2715. 10.1016/J.BIOMATERIALS.2005.11.037 16352335

[B44] Liu D. F. Li W. W. (2020). Potential-dependent extracellular electron transfer pathways of exoelectrogens. *Curr. Opin. Chem. Biol.* 59 140–146. 10.1016/J.CBPA.2020.06.005 32769012

[B45] Liu X. Gao H. Ward J. E. Liu X. Yin B. Fu T. (2020). Power generation from ambient humidity using protein nanowires. *Nature* 578 550–554. 10.1038/s41586-020-2010-9 32066937

[B46] Logan B. E. Rossi R. Ragab A. Saikaly P. E. (2019). Electroactive microorganisms in bioelectrochemical systems. *Nat. Rev. Microbiol.* 17 307–319. 10.1038/s41579-019-0173-x 30846876

[B47] Lovley D. R. (2012). Electromicrobiology. *Annu. Rev. Microbiol.* 66 391–409 10.1146/annurev-micro-092611-150104 22746334

[B48] Lovley D. R. Walker D. J. F. (2019). Geobacter Protein Nanowires. *Front. Microbiol.* 10:2078. 10.3389/fmicb.2019.02078 31608018 PMC6771412

[B49] Lovley D. R. Yao J. (2021). Intrinsically Conductive Microbial Nanowires for ‘Green’ Electronics with Novel Functions. *Trends Biotechnol.* 39 940–952. 10.1016/J.TIBTECH.2020.12.005 33419586

[B50] Malvankar N. S. Vargas M. Nevin K. P. Franks A. E. Leang C. Kim B. C. (2011). Tunable metallic-like conductivity in microbial nanowire networks. *Nat. Nanotechnol.* 6 573–579. 10.1038/nnano.2011.119 21822253

[B51] Martin D. P. Williams S. F. (2003). Medical applications of poly-4-hydroxybutyrate: A strong flexible absorbable biomaterial. *Biochem. Eng. J.* 16 97–105. 10.1016/S1369-703X(03)00040-8

[B52] Meysman F. J. R. Cornelissen R. Trashin S. Bonné R. Hidalgo-Martinez S. van der Veen J. (2019). A highly conductive fibre network enables centimetre-scale electron transport in multicellular cable bacteria. *Nat. Commun.* 10:4120. 10.1038/s41467-019-12115-7 31511526 PMC6739318

[B53] Nielsen L. P. Risgaard-Petersen N. Fossing H. Christensen P. B. Sayama M. (2010). Electric currents couple spatially separated biogeochemical processes in marine sediment. *Nature* 463 1071–1074. 10.1038/nature08790 20182510

[B54] Paterson A. F. Singh S. Fallon K. J. Hodsden T. Han Y. Schroeder B. C. (2018). Recent Progress in High-Mobility Organic Transistors: A Reality Check. *Adv. Materials* 30:e1801079. 10.1002/adma.201801079 30022536

[B55] Pfeffer C. Larsen S. Song J. Dong M. Besenbacher F. Meyer R. L. (2012). Filamentous bacteria transport electrons over centimetre distances. *Nature* 491 218–221. 10.1038/nature11586 23103872

[B56] Pirbadian S. Barchinger S. E. Leung K. M. Byun H. S. Jangir Y. Bouhenni R. A. (2014). Shewanella oneidensis MR-1 nanowires are outer membrane and periplasmic extensions of the extracellular electron transport components. *Proc. Natl. Acad. Sci. U.S.A.* 111 12883–12888. 10.1073/pnas.1410551111 25143589 PMC4156777

[B57] Prado A. G. S. Bolzon L. B. Pedroso C. P. Moura A. O. Costa L. L. (2008). Nb2O5 as efficient and recyclable photocatalyst for indigo carmine degradation. *Appl. Catalysis B* 82 219–224. 10.1016/j.apcatb.2008.01.024

[B58] Reguera G. McCarthy K. D. Mehta T. Nicoll J. S. Tuominen M. T. Lovley D. R. (2005). Extracellular electron transfer *via* microbial nanowires. *Nature* 435 1098–1101. 10.1038/nature03661 15973408

[B59] Ryder G. Zhao Houlin H. (2019). *The world’s e-waste is a huge problem. It’s also a golden opportunity. World Economic Forum Annual Meeting.* Available online at: https://www.weforum.org/agenda/2019/01/how-a-circular-approach-can-turn-e-waste-into-a-golden-opportunity/. (Accessed on Feb 8 2021)

[B60] Schauer R. Risgaard-Petersen N. Kjeldsen K. U. Tataru Bjerg J. J. Jørgensen B. Schramm A. (2014). Succession of cable bacteria and electric currents in marine sediment. *ISME J.* 8 1314–1322. 10.1038/ismej.2013.239 24451206 PMC4030233

[B61] Seidel H. Csepregi L. Heuberger A. Baumgärtel H. (1990). Anisotropic Etching of Crystalline Silicon in Alkaline Solutions: II. Influence of Dopants. *J.Electrochem. Soc.* 137 3626–3632. 10.1149/1.2086278

[B62] Sekitani T. Yokota T. Zschieschang U. Klauk H. Bauer S. Takeuchi K. (2009). Organic nonvolatile memory transistors for flexible sensor arrays. *Science* 326 1516–1519. 10.1126/science.1179963 20007895

[B63] Shi L. Dong H. Reguera G. Beyenal H. Lu A. Liu J. (2016). Extracellular electron transfer mechanisms between microorganisms and minerals. *Nat. Rev. Microbiol.* 10 651–662. 10.1038/nrmicro.2016.93 27573579

[B64] Siegenthaler K. O. Künkel A. Skupin G. Yamamoto M. (2012a). Ecoflex^®^ and ecovio^®^: Biodegradable, performance-enabling plastics. *Adv. Polymer Sci.* 245 91–136. 10.1007/12-2010-106

[B65] Siegenthaler K. O. Künkel A. Skupin G. Yamamoto M. (2012b). Ecoflex^®^ and ecovio^®^: Biodegradable, performance-enabling plastics. *Adv. Polymer Sci.* 245 91–136.

[B66] Smith A. F. Liu X. Woodard T. L. Fu T. Emrick T. Jiménez J. M. (2020). Bioelectronic protein nanowire sensors for ammonia detection. *Nano Res.* 5 1479–1484. 10.1007/S12274-020-2825-6

[B67] Stadler P. Oppelt K. Singh T. B. Grote J. G. Schwödiauer R. Bauer S. (2007). Organic field-effect transistors and memory elements using deoxyribonucleic acid (DNA) gate dielectric. *Organic Elect.* 8 648–654. 10.1016/j.orgel.2007.05.003

[B68] Summers Z. M. Fogarty H. E. Leang C. Franks A. E. Malvankar N. S. Lovley D. R. (2010). Direct exchange of electrons within aggregates of an evolved syntrophic coculture of anaerobic bacteria. *Science* 330 1413–1415. 10.1126/SCIENCE.1196526 21127257

[B69] Sure S. Ackland M. L. Torriero A. A. J. Adholeya A. Kochar M. (2016). Microbial nanowires: An electrifying tale. *Microbiology* 162 2017–2028. 10.1099/mic.0.000382 27902405

[B70] Suryana R. Khoiruddin Supriyanto A. (2013a). Beta-carotene dye of daucus carota as sensitizer on dye-sensitized solar cell. *Materials Sci. Forum* 737 15–19. 10.4028/www.scientific.net/MSF.737.15

[B71] Suryana R. Khoiruddin Supriyanto A. (2013b). Beta-carotene dye of daucus carota as sensitizer on dye-sensitized solar cell. *Materials Sci. Forum* 737 15–19. 10.4028/WWW.SCIENTIFIC.NET/MSF.737.15

[B72] Tan Y. Adhikari R. Y. Malvankar N. S. Pi S. Ward J. E. Woodard T. L. (2016). Synthetic Biological Protein Nanowires with High Conductivity. *Small* 12 4481–4485. 10.1002/smll.201601112 27409066

[B73] Tan Y. Adhikari R. Y. Malvankar N. S. War J. E. Woodard T. L. Nevin K. P. (2017). Expressing the geobacter metallireducens pila in geobacter sulfurreducens yields pili with exceptional conductivity. *mBio* 8 e02203–16. 10.1128/mBio.02203-16 28096491 PMC5241403

[B74] Thiruvallur Eachambadi R. Bonné R. Cornelissen R. Hidalgo-Martinez S. Vangronsveld J. Meysman F. J. R. (2020). An Ordered and Fail-Safe Electrical Network in Cable Bacteria. *Adv. Biosyst.* 4:2000006. 10.1002/adbi.202000006 32449305

[B75] Thiruvallur Eachambadi R. Boschker H. T. S. Franquet A. Spampinato V. Hidalgo-Martinez S. Valcke R. (2021). Enhanced Laterally Resolved ToF-SIMS and AFM Imaging of the Electrically Conductive Structures in Cable Bacteria. *Analytical Chem.* 93, 7226–7234. 10.1021/ACS.ANALCHEM.1C00298 33939426

[B76] Thorup C. Petro C. Bøggild A. Ebsen T. S. Brokjær S. Nielsen L. P. (2021). How to grow your cable bacteria: Establishment of a stable single-strain culture in sediment and proposal of Candidatus Electronema aureum GS. *Syst. Appl. Microbiol.* 44:126236. 10.1016/J.SYAPM.2021.126236 34332367

[B77] Townsend P. H. Martin S. J. Godschalx J. Romer D. R. Smith D. W. Castillo D. (1997). Silk Polymer Coating with Low Dielectric Constant and High Thermal Stability for Ulsi Interlayer Dielectric. *MRS Online Proc. Library* 1 9–17. 10.1557/PROC-476-9

[B78] Ueki T. Walker D. J. F. Tremblay P. L. Nevin K. P. Ward J. E. Woodard T. L. (2019). Decorating the Outer Surface of Microbially Produced Protein Nanowires with Peptides. *ACS Synthetic Biol.* 8 1809–1817. 10.1021/ACSSYNBIO.9B00131/SUPPL_FILE/SB9B00131_SI_001.PDF 31298834

[B79] Ueki T. Walker D. J. F. Woodard T. L. Nevin K. P. Nonnenmann S. S. Lovley D. R. (2020). An *Escherichia coli* Chassis for Production of Electrically Conductive Protein Nanowires. *ACS Synthetic Biol.* 9 647–654. 10.1021/ACSSYNBIO.9B00506/SUPPL_FILE/SB9B00506_SI_001.PDF 32125829

[B80] Valdez-Vazquez I. Robledo-Rizo J. G. Muñoz-Páez K. M. Pérez-Rangel M. de la Luz Ruiz-Aguilar G. M. (2020). Simultaneous hydrogen production and decolorization of denim textile wastewater: kinetics of decolorizing of indigo dye by bacterial and fungal strains. *Braz. J. Microbiol.* 51 701–709. 10.1007/s42770-019-00157-4 32319044 PMC7203402

[B81] Vautier M. Guillard C. Herrmann J. M. (2001). Photocatalytic Degradation of Dyes in Water: Case Study of Indigo and of Indigo Carmine. *J. Catalysis* 201 46–59. 10.1006/JCAT.2001.3232

[B82] Walker D. J. Adhikari R. Y. Holmes D. E. Ward J. E. Woodard T. L. Nevin K. P. (2018). Electrically conductive pili from pilin genes of phylogenetically diverse microorganisms. *ISME J.* 12 48–58. 10.1038/ismej.2017.141 28872631 PMC5739001

[B83] Walker D. J. F. Martz E. Holmes D. E. Zhou Z. Nonnenmann S. S. Lovley D. R. (2019). The archaellum of methanospirillum hungatei is electrically conductive. *mBio* 10 e00579–19. 10.1128/mBio.00579-19 30992355 PMC6469973

[B84] Wang F. Gu Y. O’Brien J. P. Yi S. M. Yalcin S. E. Srikanth V. (2019). Structure of Microbial Nanowires Reveals Stacked Hemes that Transport Electrons over Micrometers. *Cell* 177 361.e–369.e. 10.1016/j.cell.2019.03.029 30951668 PMC6720112

[B85] Wang Y. Rudym D. D. Walsh A. Abrahamsen L. Kim H. J. Kim H. S. (2008). *In vivo* degradation of three-dimensional silk fibroin scaffolds. *Biomaterials* 29 3415–3428. 10.1016/j.biomaterials.2008.05.002 18502501 PMC3206261

[B86] Wei Q. Mukaida M. Naitoh Y. Ishida T. (2013). Morphological Change and Mobility Enhancement in PEDOT:PSS by Adding Co-solvents. *Adv. Materials* 25 2831–2836. 10.1002/adma.201205158 23606373

[B87] Yalcin S. E. O’Brien J. P. Gu Y. Reiss K. Yi S. M. Jain R. (2020). Electric field stimulates production of highly conductive microbial OmcZ nanowires. *Nat. Chem. Biol.* 10 1136–1142. 10.1038/s41589-020-0623-9 32807967 PMC7502555

[B88] Zollinger H. (2003). Color Chemistry Syntheses, Properties, and Applications of Organic Dyes and Pigments: Von H. Zollinger; Weinheim, Basel, Cambridge, New York, VCH Verlagsgesellschaft, 1987; XII, 367 Seiten mit 40 Bildern und 16 Tabellen; Format 17 cm × 24 cm, Pappband DM. 3rd revise. Zeitschrift fü Chemie 29 259–268. 10.1002/zfch.19890291016

