# Supplementary material for: Biomaterials and Electroactive Bacteria for Biodegradable Electronics
Source: Front Microbiol. 2022 Jun 10;13:906363. doi: 10.3389/fmicb.2022.906363 (PMC9252516; doi:10.3389/fmicb.2022.906363)
Supplement: Supplementary file 1 [file Image_1.pdf]

Biomaterials and electroactive bacteria for biodegradable electronics – Supplementary material

Robin Bonné[1]* & Koen Wouters[2], Jamie J. M. Lustermans[1] and Jean V. Manca[2]

[1] Center for Electromicrobiology, Department of Biology, Aarhus University, Aarhus, Denmark
[2] X-LAB, Hasselt University, Diepenbeek, Belgium

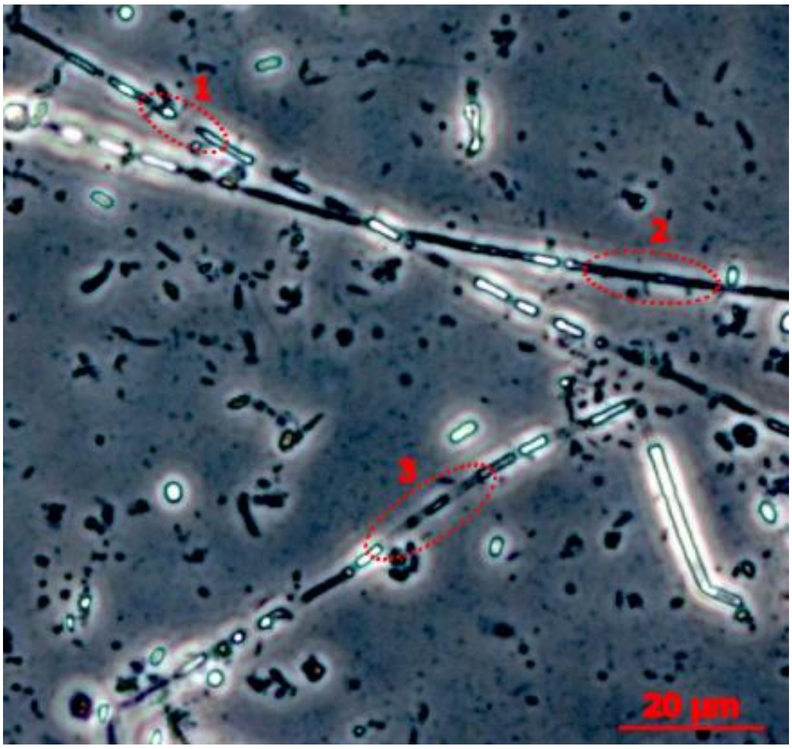

Figure S1 | Freshwater cable bacteria (*Candidatus* Electronema aureum GS) from 21-days-old sediment from the oxic zone that was used to fill a trench slide (as described in Thorup *et al*., 2021). Micrograph shows cable bacteria that have been in this slide for 9 days with a degraded cable bacterial cell (1), intact cable bacterial cells (2), and partially degraded cable bacterium where the filaments are visible as shadow lines in between remaining cell material (3).
